# Removal of Phenolic Compounds from Water Using Copper Ferrite Nanosphere Composites as Fenton Catalysts

**DOI:** 10.3390/nano9060901

**Published:** 2019-06-20

**Authors:** Carlos Moreno-Castilla, María Victoria López-Ramón, María Ángeles Fontecha-Cámara, Miguel A. Álvarez, Lucía Mateus

**Affiliations:** 1 Department of Inorganic Chemistry, Faculty of Science, University of Granada, 18071 Granada, Spain; 2 Department of Inorganic and Organic Chemistry, Faculty of Experimental Science, University of Jaen, 23071 Jaen, Spain; macamara@ujaen.es (M.Á.F.-C.); malvarez@ujaen.es (M.A.Á.); olmm0002@red.ujaen.es (L.M.)

**Keywords:** copper ferrite, cuprite, Fenton reaction, phenols, synergic effects

## Abstract

Copper ferrites containing Cu^+^ ions can be highly active heterogeneous Fenton catalysts due to synergic effects between Fe and Cu ions. Therefore, a method of copper ferrite nanosphere (CFNS) synthesis was selected that also permits the formation of cuprite, obtaining a CFNS composite that was subsequently calcined up to 400 °C. Composites were tested as Fenton catalysts in the mineralization of phenol (PHE), *p*-nitrophenol (PNP) and *p*-aminophenol (PAP). Catalysts were characterized by transmission electron microscopy (TEM), scanning electron microscopy (SEM), X-ray diffraction (XRD), Fourier transform infrared spectroscopy (FTIR), X-ray photoelectron spectroscopy (XPS) and magnetic measurements. Degradation of all phenols was practically complete at 95% total organic carbon (TOC) removal. Catalytic activity increased in the order PHE < PNP < PAP and decreased when the calcination temperature was raised; this order depended on the electronic effects of the substituents of phenols. The as-prepared CFNS showed the highest catalytic activity due to the presence of cubic copper ferrite and cuprite. The Cu^+^ surface concentration decreased after calcination at 200 °C, diminishing the catalytic activity. Cuprite alone showed a lower activity than the CFNS composite and the homogeneous Fenton reaction had almost no influence on its overall activity. CFNS activity decreased with its reutilization due to the disappearance of the cuprite phase. Degradation pathways are proposed for the phenols.

## 1. Introduction

The Fenton reaction is an advanced oxidation process (AOP) widely used to remove recalcitrant or non-biodegradable organic pollutants from waters, thanks to the formation of hydroxyl radicals (HO^•^) [1], which are powerful oxidants (E^0^ = 2.8 V). They are produced at acid pHs according to Equation (1).

Fe^2+^ + H_2_O_2_ → Fe^3+^ + OH^−^ + HO^•^ (1)

Then Fe^2+^ ions are regenerated according to Equation (2)

Fe^3+^ + H_2_O_2_ → Fe^2+^ + H^+^ + HO_2_^•^ (2)

Basically, Equations (1) and (2) describe the Fenton process, although it includes other reactions [1,2]. The reaction rate constants of Equations (1) and (2) are 70 and ~10^−2^–10^−3^ M^−1^ s^−1^, respectively [3]. Equation (2) has the lowest reaction rate and is the rate determining step in HO^•^ generation [3,4]. Therefore, the reaction given by Equation (2) must be accelerated to increase the activity of heterogeneous Fenton catalysts based on Fe ions (2). One option is to introduce Cu^+^ ions that generate hydroxyl radicals according to Equation (3).

Cu^+^ + H_2_O_2_ → Cu^2+^ + OH^−^ + HO^•^ (3)

Then Cu^2+^ ions are reduced by hydrogen peroxide [5,6,7] according to Equation (4).

Cu^2+^ + H_2_O_2_ → Cu^+^ + H^+^ + HO_2_^•^ (4)

The reaction rate constants of Equations (3) and (4) are 1.0 × 10^4^ and 4.6 × 10^2^ M^−1^ s^−1^, respectively [7], higher than those observed for Fe ions in Equations (1) and (2). Importantly, however, Fe and Cu ions may interact in bimetallic catalysts and in some copper ferrite catalysts, thus increasing their activity. This interaction has been attributed to a synergic effect between Cu^+^/Cu^2+^ and Fe^2+^/Fe^3+^ redox pairs [3,7], which can be expressed by the reaction given in Equation (5)

Fe^3+^ + Cu^+^ → Fe^2+^ + Cu^2+^ (5)

The rate constant of this reaction is 1.3 × 10^7^ M^−1^ s^−1^, which is much faster than reaction (2), producing a rapid reduction in Fe^3+^ [3]. According to these results, copper ferrites that contain Cu^+^ ions may be good heterogeneous Fenton catalysts. For this reason, the first objective of this work was to prepare copper ferrite nanosphere (CFNS) composites using a synthesis method that produces a mixture of copper ferrite and cuprite. This can be achieved by including as an ingredient ethylene glycol, which can partially reduce the cupric salt used to prepare the copper ferrite. Different portions of the as-prepared CFNS composite were calcined at 200 and 400 °C. All samples were characterized to determine their morphology, surface area, phase composition and size as well as the oxidation of the metal ions, their distribution and their magnetic properties.

The second objective was to study the activity of the CFNS composites in the mineralization of three phenols (phenol (PHE), *p*-nitrophenol (PNP), and *p*-aminophenol (PAP)) that contain substituents in their aromatic rings that possess different electron-donating and -withdrawing properties against electrophilic attacks, e.g., by hydroxyl radicals. These properties were related to the activity of the catalysts. The phenols used in this work are common water pollutants and can also be considered as model molecules for other pollutants.

## 2. Materials and Methods

### 2.1. Synthesis of CFNS Composites

A CFNS composite was prepared using a solvothermal method [8,9]. Briefly, CuCl_2_·2H_2_O (3.13 mmol) and FeCl_3_·6H_2_O (6.26 mmol) were dissolved in ethylene glycol (50 mL) followed by the addition of 4.5 g sodium acetate and 2.0 g polyethylene glycol. The mixture was vigorously stirred for 30 min and then sealed in a 125 mL Teflon-lined stainless-steel autoclave, which was heated to 200 °C and kept at this temperature for 12 h before cooling to room temperature. The black solid was centrifuged and washed several times with ethanol and finally dried at 60 °C for 8 h. It was then calcined in an air-oven at 200 and 400 °C for 3 h. Samples were kept in a desiccator until their use and were identified by adding a number to CFNS that indicated their treatment temperature (e.g., CFNS400).

### 2.2. Characterization Methods

The morphology was examined by TEM and SEM using a JEOL JEM-1010 microscope (JEOL Europe SAS, Croissy, France) and a Carl Zeiss SMT equipment (Carl Zeiss SMT, Oberkochen, Germany), respectively. Brunauer-Emmett-Teller (BET) surface area, S_BET_, was determined with N_2_ at −196 °C using an Autosorb 1 from Quantachrome (Boyton Beach, FL, USA). XPS was performed using an Escalab 200R system (Thermo Fisher Scientific, East Grinstead, UK) equipped with MgKα X-ray source as described elsewhere [6]. Previously, samples were outgassed at room temperature and 10^−7^ Pa. The crystalline structure of catalysts was examined by XRD collected on an X-ray Empyrean diffractometer with PIXcel-3D detector (Empyrean, PANanalytical, Almelo, The Netherlands) under the same experimental conditions described elsewhere [6].

FTIR spectra were collected with a Bruker Vertex 70 spectrometer (Bruker, Ettlingen, Germany) and were recorded in the range of 1800–400 cm^−1^ with 2 cm^−1^ resolution in transmission mode, at room temperature and with attenuated total reflectance accessories.

The magnetization (M) of catalysts versus the magnetic field applied (H) at room temperature were collected with a SQUID magnetometer (Quantum Design model MPMS-XL, San Diego, CA, USA). The saturation and remnant magnetization, M_S_ and M_R_, respectively, and the coercivity, H_C_, of the bulk samples were obtained from the M–H curves.

### 2.3. Fenton Reaction

The Fenton reaction was carried out at 35 °C and pH 3 using different conical flasks containing 0.1 L of reaction solution and 100 mg L^−1^ of catalyst that were shaken at 300 rpm. The concentration of the phenolic compounds was always 0.107 mM and that of the H_2_O_2_ was 1.50, 1.45 and 1.40 mM for PHE, PNP and PAP, respectively, and these concentrations correspond to the stoichiometric mineralization of the phenolic compound used according to Equations (6)–(8).

C_6_H_6_O + 14H_2_O_2_ → 6CO_2_ + 17H_2_O (6)

2C_6_H_5_NO_3_ + 27H_2_O_2_ → 12CO_2_ + 32H_2_O + 2NO_2_ (7)

C_6_H_7_NO + 13H_2_O_2_ → 6CO_2_ + 15H_2_O + NH_3_ (8)

The experimental procedure was similar to that described elsewhere [10]. Phenol compound concentrations in solution were determined using a Thermo-Fisher HPLC equipped with an UV8000 photodiode detector, and a Hypersil Gold chromatographic column (250 × 4.6 mm). The mobile phase for PHE and PNP was a mixture of 60% HPLC grade methanol and 40% ultrapure water in isocratic mode with an elution flow rate of 0.8 mL min^−1^; in the case of PAP, the mobile phase was a mixture of 48% HPLC grade methanol and 52% ultrapure water in isocratic mode (0.5% formic acid) with an elution flow rate of 1 mL min^−1^. The injection volume was 20 μL in all samples. The detector wavelength was 269, 318 and 220 nm for PHE, PNP and PAP, respectively. The TOC was measured with a TOC-5000A model Shimadzu analyzer, considering the average of at least three measurements with an accuracy of ±5%.

The residual H_2_O_2_ concentration in solution and the leaching of copper and iron from the catalysts was determined as described elsewhere [6]. Concentrations of NO_2_^−^, NO_3_^−^ and NH_4_^+^ ions in solution were determined with Specific Merck Spectroquant kits, based on the formation of a red-violet azo dye for NO_2_^−^ ions (Test kit 1.14776), a red nitro compound for NO_3_^−^ ions (Test kit 1.14773) and an indophenol blue derivative for NH_4_^+^ ions (Test kit 1.14752). These compounds were determined photometrically with a Palintest 7100 Photometer at 542, 330 and 690 nm, for NO_2_^−^, NO_3_^−^ and NH_4_^+^ ions, respectively.

## 3. Results and Discussion

### 3.1. Characteristics of the Catalysts

**Figure 1:**
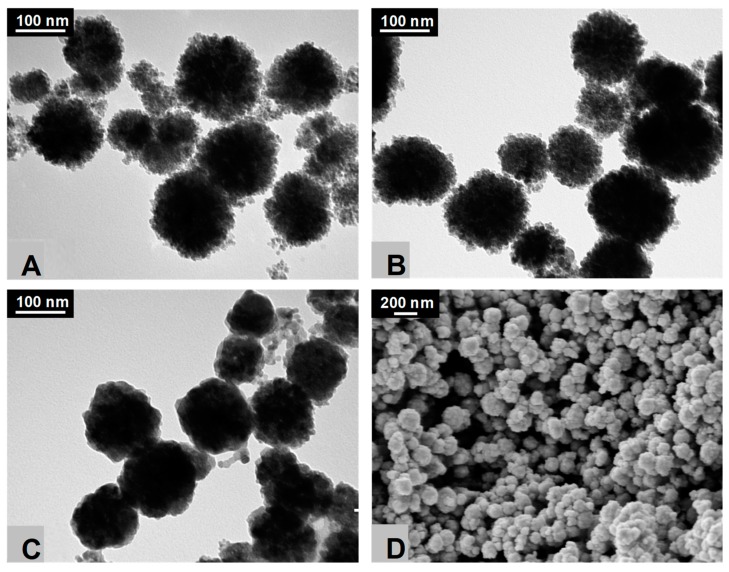
TEM micrographs of (**A**) copper ferrite nanosphere (CFNS); (**B**) CFNS200; (**C**) CFNS400 and SEM micrograph of (**D**) CFNS400.

Figure 1 depicts TEM and SEM micrographs, showing that all CFNS samples are composed of nanospheric particles. The CFNS and CFNS200 samples consisted of numerous well-distributed heterojunction nanocrystals with a dandelion-like structure. This structure disappeared in sample CFNS400 because the increase in calcination temperature sintered the nanocrystals, smoothing the surface of the nanospheres. However, the size of the CFNS was not affected by calcination. Thus, the mean diameter of all samples, based on at least 200 particles from different images, was around 150 nm. Sintering of the nanocrystals on the surface of the nanospheres at higher temperatures reduced the S_BET_ from 47 m^2^ g^−1^ in CFNS to 38 and 12 m^2^ g^−1^ in CFNS200 and CFNS400, respectively.

**Figure 2:**
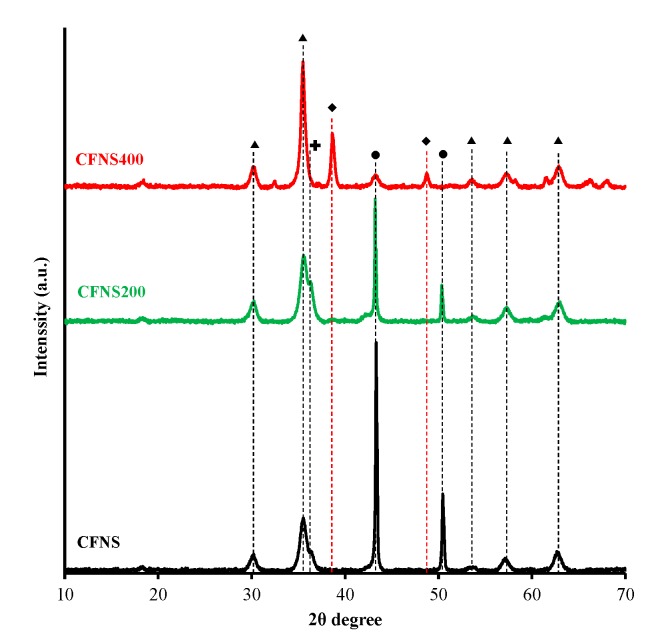
XRD patterns of CFNS composites. (▲) cubic spinel; (♦) tenorite; (●) copper; (✚) cuprite.

Figure 2 depicts the XRD patterns of the fresh catalysts, and Table 1 exhibits the results obtained. CFNS and CFNS200 showed the same phases: c-CuFe_2_O_4_, Cu_2_O and Cu. The presence of metallic Cu and Cu^+^ ions in CFNS was due to the reduction by ethylene glycol of some Cu^2+^ ions present in the solution. CFNS200 showed similar spinel content and crystallite size to those of CFNS but a higher Cu_2_O content and lower Cu content, due to the oxidation of part of the metallic Cu during calcination at 200 °C [11,12], which also increased the crystallite size. Cuprite and metallic copper were oxidized to tenorite at 400 °C (CFNS400). The phase percentage of c-CuFe_2_O_4_ slightly decreased from CNFS to CNFS400 but the crystallite size remained unchanged.

**Figure 3:**
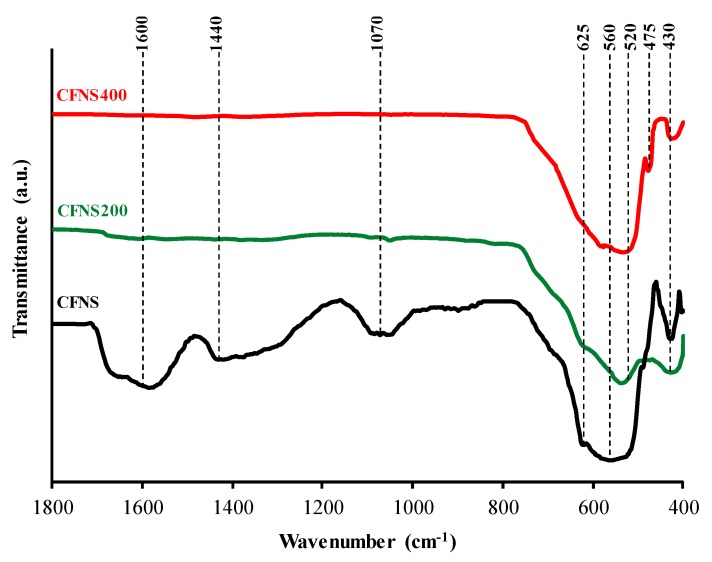
FTIR spectra of CFNS composites.

FTIR spectra between 1800 and 400 cm^−1^ for the CFNS sample and its calcined derivatives are depicted in Figure 3. CFNS shows a broad absorption band at ~1600 cm^−1^, assigned to the bending mode of remaining H_2_O molecules and OH groups [13,14]; the spectrum also shows a band at ~1440 cm^−1^, assigned to C–H bending of methylene groups from the compounds used to prepare this sample and at ~1070 cm^−1^, attributed to O–H stretching modes [15]. All of these bands disappeared in the calcined samples demonstrating the complete removal of organic residues and water.

FTIR spectra of the original and calcined CNFS samples showed principal absorption bands in the range of ~600 and ~400 cm^−1^, typical of ferrites and corresponding to the intrinsic lattice vibration of tetrahedral A sites (lesser bond length of oxygen-metal ions) and octahedral B sites (greater bond length), respectively [16,17,18]. In inverse ferrites, such as copper ferrite, the tetrahedral and octahedral sites would be occupied by Fe^3+^ and Cu^2+^ ions, respectively.

CFNS shows vibration bands at 625 and 560 cm^−1^, assigned to Cu^2+^–O^2−^ and Fe^3+^–O^2−^ vibrations in tetrahedral sites, respectively, and also at 430 cm^−1^, assigned to Cu^2+^ –O^2−^ vibrations in octahedral sites [19,20]. Therefore, Cu^2+^ ions occupy both octahedral and tetrahedral sites. Sample CFNS200 also shows bands at 625, 530 and 430 cm^−1^. On the other hand, the band at 625 cm^−1^ might also be attributed to Cu^+^–O^2−^ vibrations in Cu_2_O [21,22], which was shown by XRD to be present in CNFS and CNFS200 (Table 1).

In CFNS400 the band at 625 cm^−1^ disappears, the band assigned to Fe^3+^–O^2−^ vibrations in tetrahedral sites is shifted to 520 cm^−1^, and the band at 430 cm^−1^ did not vary. XRD revealed that CFNS400 contained CuO, which shows peaks at 588, 530, 480 and 430 cm^−1^ [21,22]. Some of them overlapped with other peaks assigned to the spinel.

**Figure 4:**
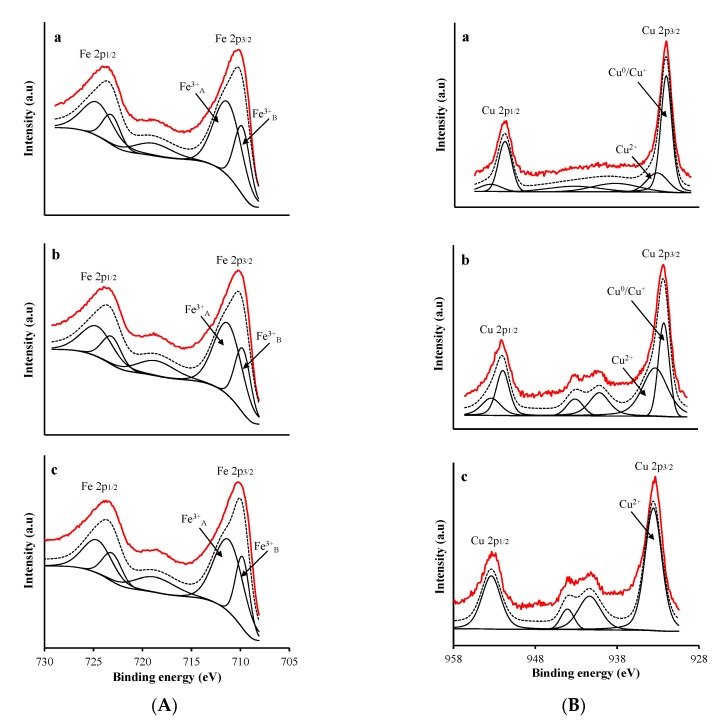
XPS profiles of (**A**) Fe 2p and (**B**) Cu 2p region in: (a) CFNS; (b) CFNS200; (c) CFNS400. Continuous red line: experimental profile; discontinuous black line: fitted profile.

Figure 4A,B depicts the XP spectra of Fe and Cu 2p core level regions, respectively, and the results are compiled in Table 2. Deconvolution of the broad Fe 2p_3/2_ peak showed two more peaks: the first at a BE of 709.8 eV accompanied by a satellite peak at 718.5 eV, indicating the presence of Fe^3+^ cations [6,23]; and the second at 711.3 eV indicating the presence of Fe^3+^ cations in two different coordinations, i.e., tetrahedral sites at higher BE (second peak) and octahedral sites at lower BE (first peak) [24]. 

The Cu 2p_3/2_ spectra of CFNS and CFNS200 showed two peaks at 932.3 and 933.5 eV, assigned to reduced copper species (Cu^0^/Cu^+^) and Cu^2+^, respectively, and a shake-up satellite at around 942 eV [25,26]. The Cu^+^ surface concentration was lower on CFNS200 than on CFNS (Table 2), consistent with the larger crystallite size of Cu_2_O measured by XRD in the former. The Cu 2p_3/2_ spectra of CFNS400 had only one peak at 933.6 eV, corresponding to Cu^2+^.

**Figure 5:**
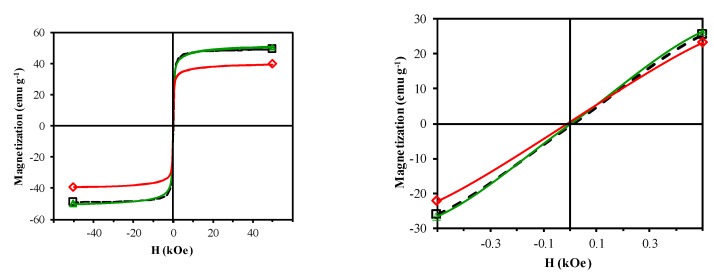
Magnetization versus applied magnetic field for: (**□**) CFNS; (△) CFNS200; (◇) CFNS400.

Figure 5 depicts the room temperature M–H curves, which have a normal S-shape, suggesting a significant content of ferrimagnetic materials. Table 3 displays the results derived from these curves. All samples can be considered superparamagnetic due to their very low M_R_/M_S_ ratio [27]. The saturation magnetization is influenced by the crystallite size of the ferrimagnetic phase and the distribution of Cu^2+^ ions between B and A sites, i.e., the inversion degree of the spinel. Thus, an increase in crystallite size and inversion degree increases the M_S_ value [27,28]. CFNS and CFNS200 showed similar M_S_ values because they have a similar percentage and crystallite size of the ferrimagnetic spinel phase. CFNS400 showed a lower M_S_ value due to a decrease in the percentage of the spinel phase.

### 3.2. Fenton Reaction

**Figure 6:**
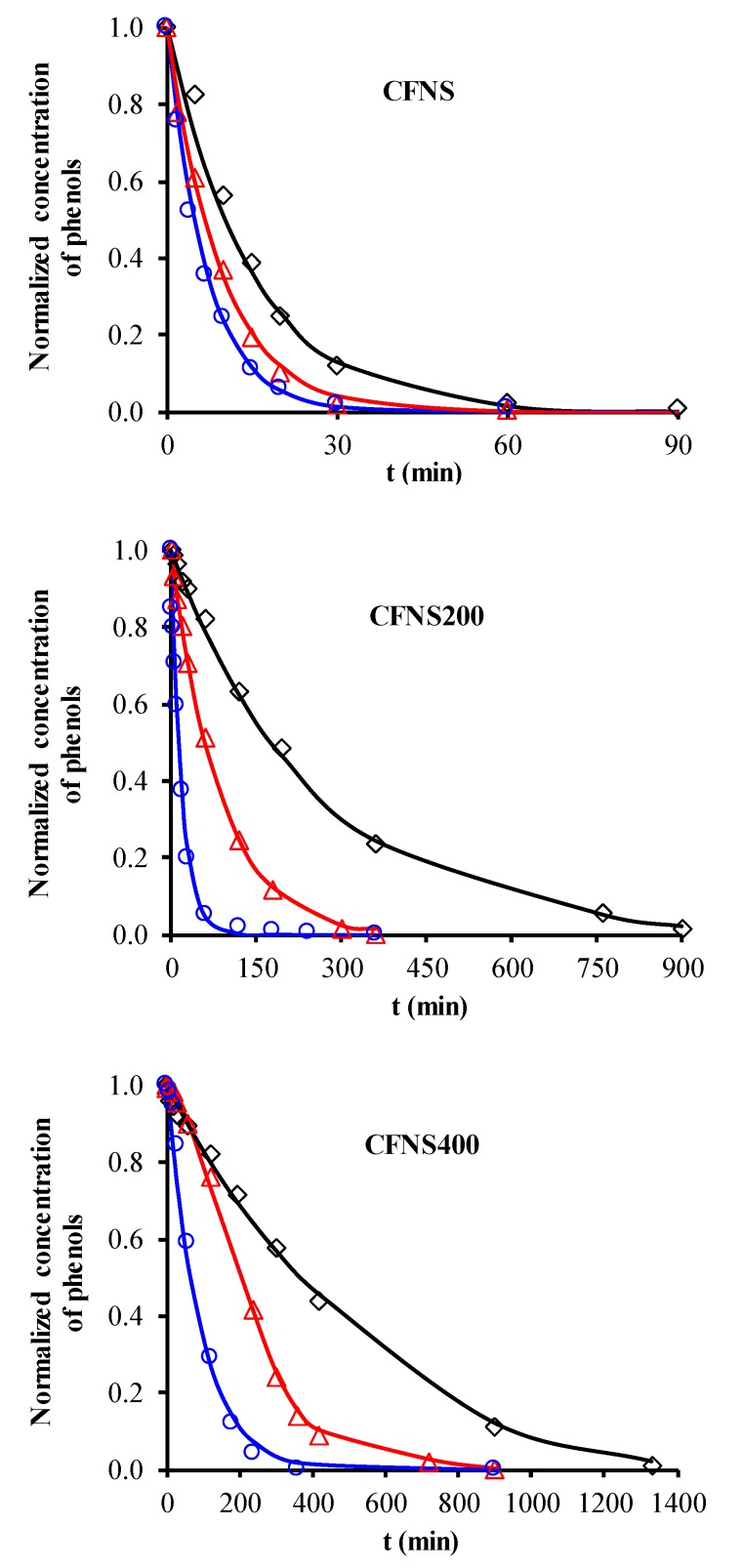
Degradation kinetics of phenols: (◇) phenol (PHE); (△) *p*-nitrophenol (PNP); (○) *p*-aminophenol (PAP). Reaction conditions: T = 35 °C; Mass of catalyst = 100 mg L^−1^, C_phenols_ = 0.107 mM, CH_2_O_2__(PHE)_ = 1.50 mM, CH_2_O_2__(PNP)_ = 1.45 mM, CH_2_O_2__(PAP)_ = 1.40 mM, pH 3, V = 0.1 L.

**Figure 7:**
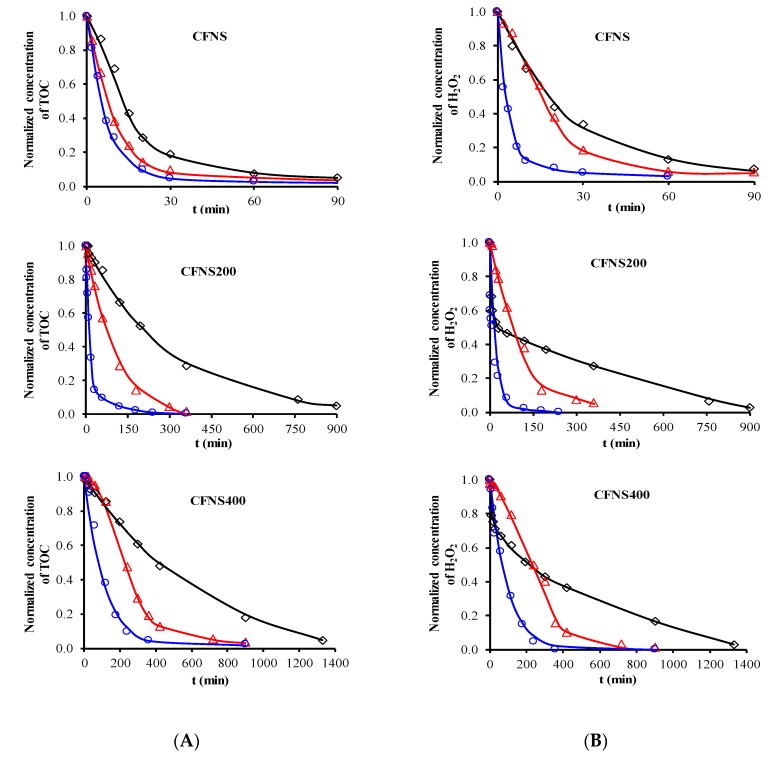
(**A**) Kinetics of total organic carbon (TOC) removal and (**B**) kinetics of H_2_O_2_ decomposition: (◇) PHE; (△) PNP; (○) PAP. Reaction conditions: T = 35 °C; Mass of catalyst = 100 mg L^−1^, C_phenols_ = 0.107 mM, CH_2_O_2__(PHE)_ = 1.50 mM, CH_2_O_2__(PNP)_ = 1.45 mM, CH_2_O_2__(PAP)_ = 1.40 mM, pH 3, V = 0.1 L.

**Figure 8:**
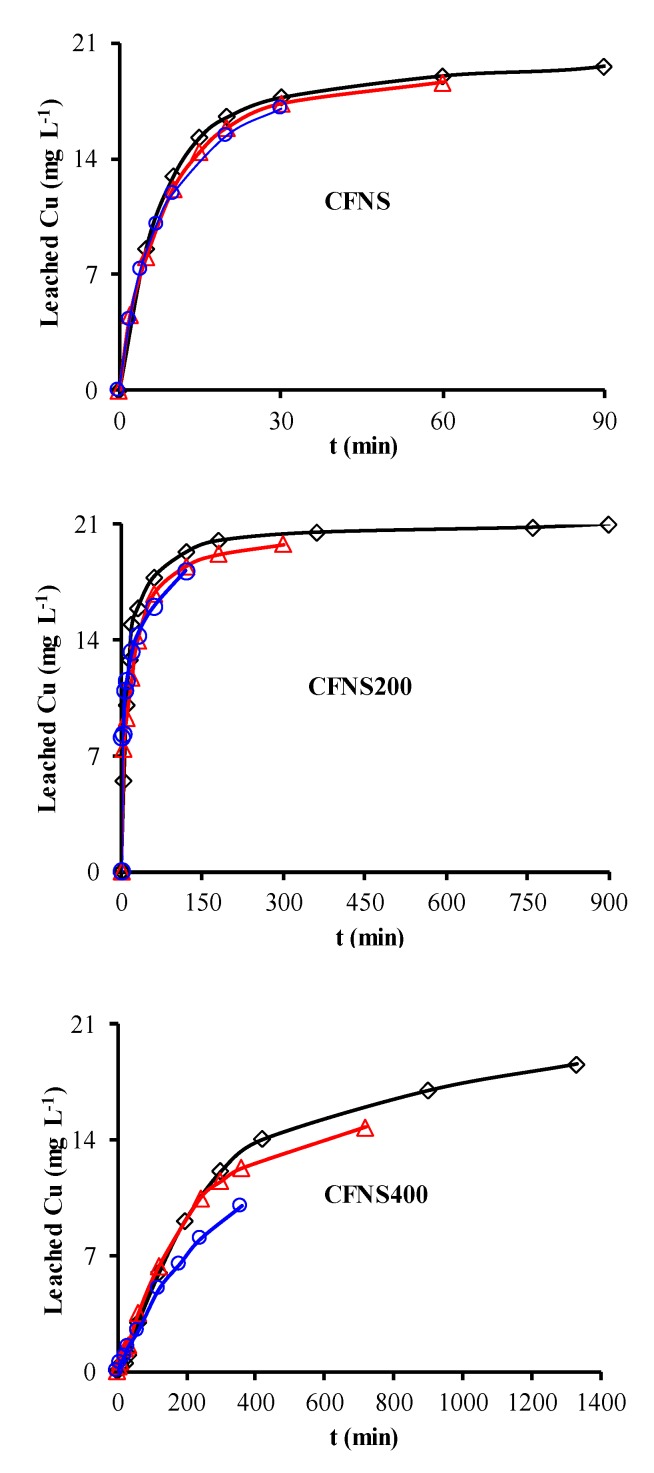
Variations in the concentration of leached Cu ions at 35 °C from CFNS composites for different phenols: (◇) PHE; (△) PNP; (○) PAP.

Figure 6 depicts the PHE, PNP and PAP degradation kinetics curves obtained with different catalysts. Similar curves were obtained for TOC removal (Figure 7A) and H_2_O_2_ decomposition (Figure 7B). Figure 8 plots the Cu ions leached from the catalysts against reaction time. Table 4 exhibits the results obtained from these curves when 95% TOC removal was reached. The degradation of phenols using H_2_O_2_ alone, with no catalyst, was 1.9%, 2.5% and 7.5% for PHE, PNP and PAP, respectively, indicating the poor oxidation power of H_2_O_2_ against these phenols. In addition, blank experiments in the absence of H_2_O_2_ showed no adsorption of PHE or PAP on the catalyst and only a slight adsorption of PNP (~1.4%), demonstrating that the adsorption of phenols on these catalysts is negligible.

According to the results in Table 4, degradation of the phenols was practically complete at 95% TOC removal. The activity of the catalysts, given by the values of t and k_d_, depended on the type of phenol and the calcination temperature, increasing in the order PHE < PNP < PAP and decreasing at higher calcination temperatures. This order can be explained by the electron-donating (–OH and –NH_2_) or –withdrawing properties (–NO_2_) of the substituents of the phenols against electrophilic attack, e.g., by hydroxyl radicals. Thus, two, four and six positions of the aromatic ring are activated in PHE; two and six positions are doubly activated, and the fourth position is singly in PNP; and all positions are activated in PAP. Therefore, the above order of catalytic activity matches the order of increase in the number of activated aromatic ring positions.

CFNS has the highest catalytic activity due to the presence of cuprite together with cubic copper ferrite. Cu^+^ is known to be a good catalyst to decompose H_2_O_2_ into hydroxyl radicals [6,7], and a synergistic effect between Cu and Fe species was recently observed in Cu-Fe bimetallic catalysts [3]. This is because Cu^+^ produces a major increase in the rate constant for the reduction of Fe^3+^ to Fe^2+^ (Equation (5)), thereby enhancing the generation of hydroxyl radicals and the phenol degradation efficiency. Importantly, in comparison to CFNS200, CFNS has the same percentage and crystallite size of cubic spinel (Table 1) and a lower cuprite content but shows a higher activity, attributable to its smaller Cu_2_O crystallite size and therefore higher Cu^+^ surface concentration (Table 2).

Given the presence of cuprite in the above catalysts, its catalytic activity to degrade the phenols was tested using a commercial cuprite under the same experimental conditions as for CFNS and using the same amount of cuprite 17.2 mg L^−1^ as in this catalyst (see Table 1). As shown in Table 4, the percentage of PHE, PNP and PAP degraded at time t was, respectively, 18%, 52% and 55% lower than obtained with CFNS, especially in the case of PHE. These results demonstrate the synergistic effect between Cu and Fe species in the CFNS catalyst.

The TOC/H_2_O_2_ weight ratio, a measure of the efficiency of H_2_O_2_ utilization for 95% TOC removal, was not influenced by the catalyst used but rather by the type of phenol degraded, being ~0.30 for PNP and ~0.19 for PHE and PAP (Table 4).

Cu ions leached from all catalysts (Table 4), decreasing in the order PHE > PNP > PAP. The amount of Cu leached did not depend on the type of phenol but rather on the reaction time needed to reach 95% TOC removal, i.e., the contact time of the catalyst with the reactive solution. A smaller amount of Cu leached from CFNS400 than from the other two catalysts. No Fe ions leached from any of these catalysts.

Cu ions leached can act as homogeneous Fenton catalysts. To check this possibility, the homogeneous reaction was conducted using the amount of Cu ions leached from the CFNS catalyst at 95% TOC removal for each phenolic compound (see Table 4). Cu ion concentrations were added as sulphate. Table 4 shows that the percentage degradation for the CFNS catalyst at time t was 1, 3 and 13% for PHE, PNP and PAP, respectively, the same order of increase as observed for the heterogeneous Fenton reaction. These low percentages mean that the homogeneous Fenton reaction has practically no influence on the overall activity of the CFNS catalyst.

NO_3_^−^ and NO_2_^−^ ions were detected in the PNP degradation and NH_4_^+^ ions in the corresponding PAP degradation, and the results obtained (Table 4) were used to determine the percentage of nitrogen removed. This value was 21% for PNP and 86% for PAP with CFNS, versus 25% for PNP and 88% for PAP with CFNS400. The larger amount of N removed from the PAP solution is attributable to the formation of ammonia, which can escape more easily from the solution into the atmosphere. In addition, N removal was slightly higher with increased calcination temperature of the catalyst.

**Figure 9:**
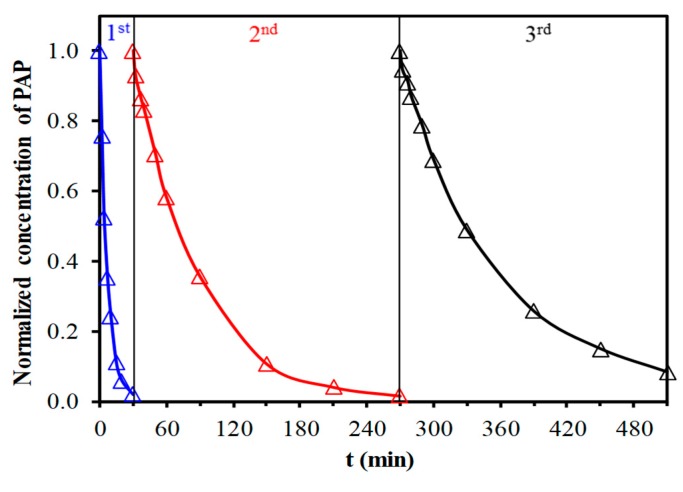
PAP degradation kinetics with fresh and reutilized CFNS catalyst.

Degradation of the most active phenolic compound, PAP, was also studied using the recycled CFNS catalyst. For this purpose, the catalyst was withdrawn by filtration after its first use, washed with distilled water to neutral pH, and then used again in the PAP removal. This process was repeated twice, determining the PAP degradation kinetics (Figure 9) and Cu ion leaching. The kinetics decreased with the reutilization of the catalysts. Thus, the pseudo-first order rate constant for the degradation was 0.145, 0.018 and 0.012 min^−1^ and the Cu ion leaching was 17.0, 5.1 and 0.2 mgL^−1^ for the fresh catalyst and its first and second reutilizations, respectively. These results are attributed to the disappearance of the cuprite phase from CFNS after each reutilization [6] and would explain the major decrease in the activity of the catalyst after its first use.

HPLC was used to study the degradation intermediates of the phenols after treatment with the CFNS catalyst. Table 5, Table 6 and Table 7 exhibit the results obtained for PHE, PNP and PAP at two reaction times as a function of the activity of the phenolic compound. The appearance of these reaction intermediates can be explained by taking account of the activated aromatic ring positions against HO^•^ electrophilic attack and results published in the literature [29,30,31,32,33,34].

**Figure 10:**
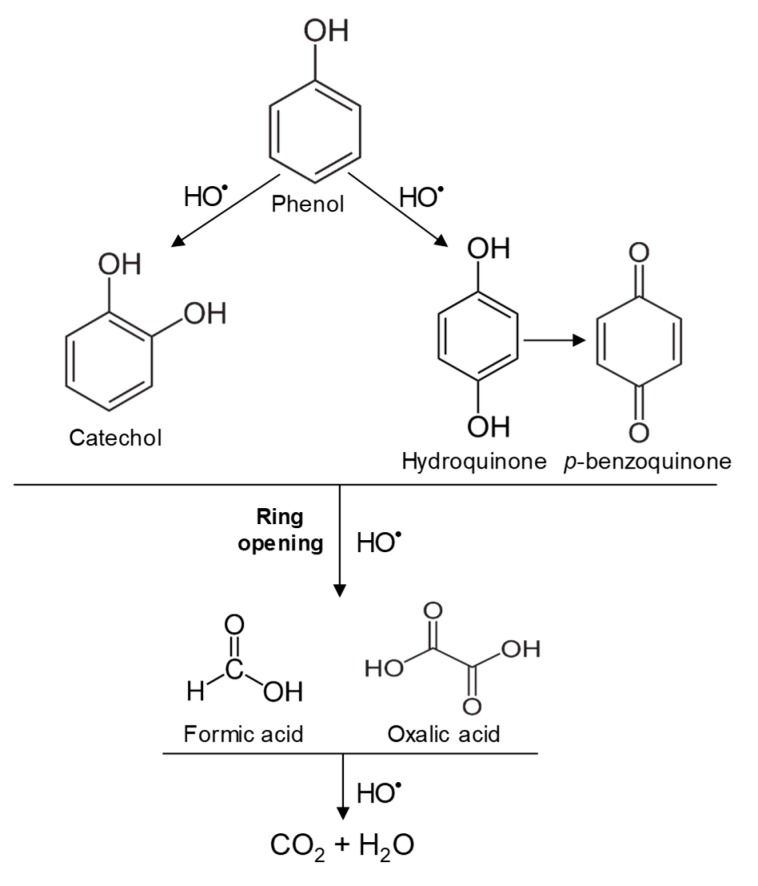
Proposed PHE degradation pathway.

In the case of PHE, PHE, catechol, hydroquinone, *p*-benzoquinone, oxalic and formic acids were observed at 60 min of reaction time; the catechol and hydroquinone comes from the HO^•^ attack on positions two and four, respectively, which are activated; *p*-benzoquinone comes from the oxidation of hydroquinone; and oxalic and formic acids from the ring opening of the aromatic compounds. Catechol and the acidic compounds remain at 90 min of reaction time; catechol is still present at this level of TOC removal because it is highly resistant to ring opening. The possible PHE degradation pathway is depicted in Figure 10.

**Figure 11:**
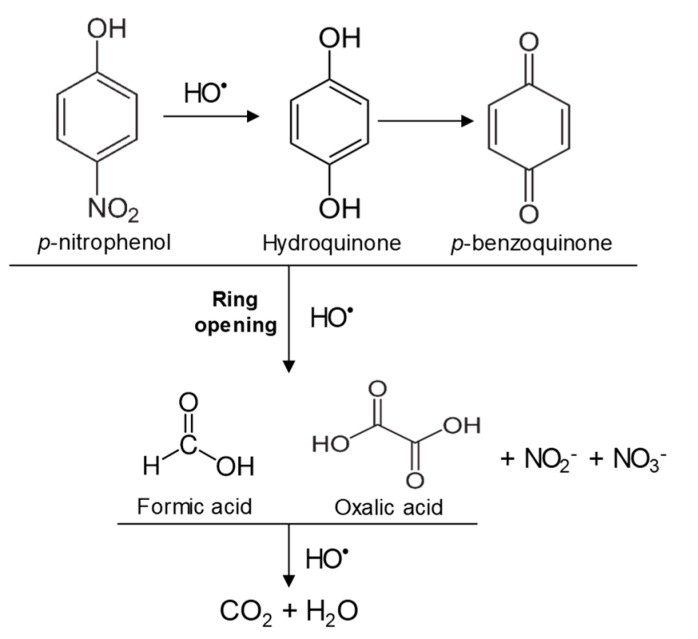
Proposed PNP degradation pathway.

In the case of PNP, PNP, hydroquinone, *p*-benzoquinone, oxalic and formic acids were observed at 30 min of reaction time. Aromatic ring position four is activated and positions two and six are doubly activated; therefore, hydroquinone is obtained after substitution of NO_2_^−^ by a phenol group, and its subsequent oxidation yields *p*-benzoquinone. The oxalic and formic acids come from ring opening of the aromatic compounds against HO^•^ electrophilic attack. Both acids remained after 60 min of reaction time. The possible PNP degradation pathway is depicted in Figure 11.

**Figure 12:**
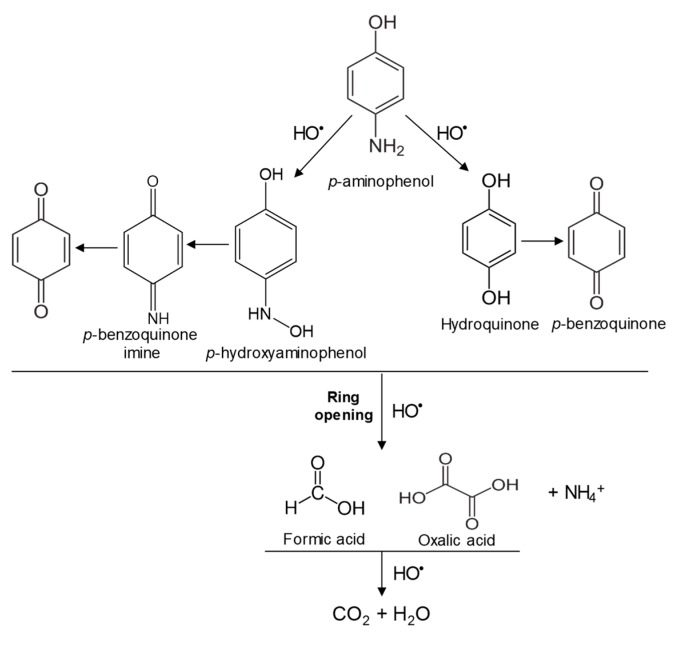
Proposed PAP degradation pathway.

In the case of PAP, PAP, hydroquinone, *p*-benzoquinone, *p*-benzoquinone imine, oxalic and formic acids appeared after 10 min of reaction time; all aromatic ring positions are activated by the HO^•^ attack. Therefore, hydroquinone comes from the substitution of the amino group by the phenol group and *p*-benzoquinone imine from the HO^•^ attack on the amino group. The subsequent oxidation of these compounds yields *p*-benzoquinone. As noted above, the oxalic and formic acids come from ring opening of the aromatic compounds. *P*-benzoquinone imine and both acids remained after 30 min of reaction time. The possible PAP degradation pathway is depicted in Figure 12.

## 4. Conclusions

CFNS samples are composed of nanospheric particles. CFNS and CFNS200 contain three phases: c-CuFe_2_O_4_, Cu_2_O and Cu. Cuprite and metallic copper were oxidized to CuO when the calcination temperature increased to 400 °C. XPS showed that the Cu^+^ surface concentration was lower on CFNS200 than on CFNS due to the larger crystallite size of Cu_2_O as measured by XRD. All samples had very low M_R_/M_S_ ratios, indicating substantial superparamagnetic behavior.

The activity of the catalysts decreased with higher calcination temperature and increased in the order PHE < PNP < PAP, matching the order of increase in the number of activated aromatic ring positions against electrophilic attacks. CFNS has the highest catalytic activity due to the presence of cuprite along with cubic copper ferrite. The phases of CFNS200 were similar to those of CFNS but its catalytic activity was lower, attributable to the lower Cu^+^ surface concentration caused by its larger Cu_2_O crystallite size. The cuprite alone showed lower catalytic activity than the CFNS composite, indicating the synergic effect between cuprite and copper ferrite. Cu ion leaching depended on the reaction time. The homogeneous Fenton reaction (due to leached Cu ions) had practically no influence on the overall activity of the CFNS catalyst. PAP degradation and Cu ion leaching decreased with reutilization of the CFNS catalyst, attributable to the disappearance of the cuprite phase after each reutilization. Possible degradation pathways are proposed for these phenols based on their degradation intermediates.

## Figures and Tables

**Table 1:** Crystalline phases and percentages, and crystallite sizes of the catalysts from XRD patterns.

Sample	Crystalline Phase	Phase Percentage	Crystalline Size (nm)
CFNS	Cubic spinel (c-CuFe_2_O_4_)	67.5	7.8
	Cuprite (Cu_2_O)	17.1	9.8
	Copper	15.4	90.0
CFNS200	Cubic spinel (c-CuFe_2_O_4_)	66.7	7.9
	Cuprite (Cu_2_O)	20.1	13.8
	Copper	13.2	110.1
CFNS400	Cubic spinel (c-CuFe_2_O_4_)	62.0	8.3
	Tenorite (CuO)	38.0	24.1

**Table 2:** Binding energy (eV) of the main XPS peaks with percentage (in parentheses) and Cu/Fe surface atomic ratio.

Sample	Fe 2p_3/2_	Fe_at_	Cu 2p_3/2_	Cu_at_	Cu/Fe
	eV	%	eV	%	
CFNS	709.8 (35)	24.7	932.3 (78)	5.4	0.22
	711.3 (65)		933.5 (22)		
CFNS200	709.8 (33)	24.4	932.3 (41)	5.0	0.21
	711.3 (67)		933.5 (59)		
CFNS400	709.8 (32)	23.5	933.6 (100)	4.7	0.20
	711.2 (68)				

**Table 3:** Saturation magnetization (M_S_), remnant magnetization (M_R_), and coercivity (H_C_) values of the catalysts.

Sample	M_S_	M_R_	M_R_/M_S_	H_C_
	emu g^−1^			Oe
CFNS	49.20	0.78	0.02	15
CFNS200	50.10	0.35	0.01	7
CFNS400	39.71	0.50	0.01	10

**Table 4:** Results obtained at the time needed to reach 95% TOC removal (t), percentage of PHE, PNP and PAP degraded, pseudo-first order rate constant for degradation (k_d_), TOC_removed_/H_2_O_2decomposed_ weight ratio (TOC/H_2_O_2_), Cu ion leaching (Cu_leac._) and concentration of NO_3_^−^, NO_2_^−^ and NH_4_^+^ ions.

Catalyst	Pollutant	T	Degraded	k_d_	TOC/H_2_O_2_	Cu_leac._	NO_3_^−^	NO_2_^−^	NH_4_^+^
		min	%	min^−1^		mg L^−1^	mM		
CFNS	PHE	90	99.0	0.068	0.20	19.6	---	---	---
	PNP	60	99.7	0.106	0.31	18.6	0.073	0.012	0.000
	PAP	30	98.0	0.145	0.19	17.0	0.000	0.000	0.015
CFNS200	PHE	900	98.4	0.004	0.19	21.0	n.d. *	n.d.	n.d.
	PNP	300	98.3	0.012	0.31	20.3	n.d.	n.d.	n.d.
	PAP	120	98.3	0.051	0.19	18.2	n.d.	n.d.	n.d.
CFNS400	PHE	1330	99.1	0.002	0.19	18.6	---	---	---
	PNP	720	98.0	0.004	0.30	14.8	0.075	0.005	0.000
	PAP	360	99.7	0.011	0.18	10.0	0.000	0.000	0.013

* n.d. = non-determined.

**Table 5:** Intermediate compounds from PHE degradation at different reaction times and TOC removal.

Compounds	Structural Formula	60 min—71.4%	90 min—95%
Phenol	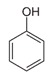	√	
Catechol	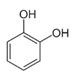	√	√
Hydroquinone	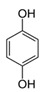	√	
*p*-benzoquinone	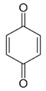	√	
Oxalic acid	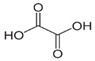	√	√
Formic acid	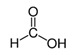		√

**Table 6:** Intermediate compounds from PNP degradation at different reaction times and TOC removal.

Compounds	Structural Formula	30 min—90.5%	60 min—95%
*p*-nitrophenol	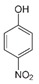	√	
Hydroquinone	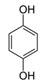	√	
*p*-benzoquinone	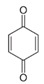	√	
Oxalic acid	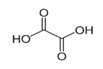	√	√
Formic acid	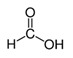	√	√

**Table 7:** Intermediate compounds from PAP degradation at different reaction times and TOC removal.

Compounds	Structural Formula	10 min—71.4%	30 min—95%
*p*-aminophenol	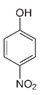	√	
Hydroquinone	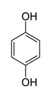	√	
*p*-benzoquinone	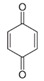	√	
*p*-benzoquinone imine	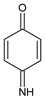	√	√
Oxalic acid	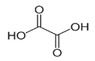	√	√
Formic acid	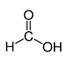	√	√
